# Performance of Legiolert Test vs. ISO 11731 to Confirm *Legionella pneumophila* Contamination in Potable Water Samples

**DOI:** 10.3390/pathogens9090690

**Published:** 2020-08-23

**Authors:** Maria Scaturro, Matteo Buffoni, Antonietta Girolamo, Sandra Cristino, Luna Girolamini, Marta Mazzotta, Maria Antonietta Bucci Sabattini, Cristina Maria Zaccaro, Leonarda Chetti, Microbiology Arpa Novara Laboratory, Antonino Bella, Maria Cristina Rota, Maria Luisa Ricci

**Affiliations:** 1 Department Infectious Diseases, National Institute of Health, Viale Regina Elena 299, 00161 Rome, Italy; maria.scaturro@iss.it (M.S.); buffoni.matteo@gmail.com (M.B.); antonietta.girolamo@iss.it (A.G.); antonino.bella@iss.it (A.B.); mariacristina.rota@iss.it (M.C.R.); 2 Department of Biological, Geological, and Environmental Sciences, University of Bologna, via San Giacomo 12, 40126 Bologna, Italy; sandra.cristino@unibo.it (S.C.); luna.girolamini2@unibo.it (L.G.); marta.mazzotta2@unibo.it (M.M.); 3 Reference Laboratory for Environmental Control, Health Protection Agency, Bologna, Emilia Romagna Region, Via Trachini, 17, 40138 Bologna, Italy; mbucci@arpae.it (M.A.B.S.); cmzaccaro@arpae.it (C.M.Z.); lchetti@arpae.it (L.C.); 4 Reference Laboratory for Environmental Control, Health Protection Agency, Novara, Piemonte Region, Viale Roma 7/e, 28100 Rome, Italy; legionella@arpa.piemonte.it

**Keywords:** *Legionella*, Legiolert, ISO 11731, plate culture, potable water samples

## Abstract

Detection and enumeration of *Legionella* in water samples is of great importance for risk assessment analysis. The plate culture method is the gold standard, but has received several well-known criticisms, which have induced researchers to develop alternative methods. The purpose of this study was to compare *Legionella* counts obtained by the analysis of potable water samples through the plate culture method and through the IDEXX liquid culture Legiolert method. *Legionella* plate culture, according to ISO 11731:1998, was performed using 1 L of water. Legiolert was performed using both the 10 mL and 100 mL Legiolert protocols. Overall, 123 potable water samples were analyzed. Thirty-seven (30%) of them, positive for *L. pneumophila*, serogroups 1 or 2–14 by plate culture, were used for comparison with the Legiolert results. The Legiolert 10 mL test detected 34 positive samples (27.6%) and the Legiolert 100 mL test detected 37 positive samples, 27.6% and 30% respectively, out of the total samples analyzed. No significant difference was found between either the Legiolert 10 mL and Legiolert 100 mL vs. the plate culture (*p* = 0.9 and *p* = 0.3, respectively) or between the Legiolert 10 mL and Legiolert 100 mL tests (*p* = 0.83). This study confirms the reliability of the IDEXX Legiolert test for *Legionella pneumophila* detection and enumeration, as already shown in similar studies. Like the plate culture method, the Legiolert assay is also suitable for obtaining isolates for typing purposes, relevant for epidemiological investigations.

## 1. Introduction

*Legionella* is a genus consisting of fastidious waterborne pathogens responsible for a severe form of pneumonia named Legionnaires’ disease (LD) and for a flu-like infection known as Pontiac fever (PF) [1]. *Legionella* is widespread in natural freshwater environments, where it can be found free-living or intracellularly in hosts such as amoebae [2]. Among the 62 species known to date, *L*. *pneumophila* is the species most frequently found in cases of infection, amounting in 2018 to approximately 94.1% of the culture-confirmed LD cases in notified in EU/EEA (European Legionnaires’ disease Surveillance Network annual meeting 2019, unpublished data). However, just under half of the known species cause illness and, in several countries such as New Zealand, soil-born *L. longbeachae* is the primary cause of LD [1]. Infection is acquired through inhalation of contaminated aerosols produced by various man-made water systems, such as showers, spa pools, fountains and cooling towers of air conditioning systems [3]. When *Legionella* colonizes the water systems, it often finds favorable conditions for growth, such as temperatures between 25 °C and 45 °C or the presence of biofilm, reaching high concentrations and becoming a serious risk for human health. After the first LD outbreak occurred in Philadelphia in 1976, numerous other outbreaks and sporadic cases have been reported worldwide [4,5,6,7,8,9,10]. In Italy in 2018, the incidence of Legionnaires’ disease was 4.9 cases per million inhabitants, 2964 notified cases [11], and in the European network for Legionnaires’ disease surveillance, Italy ranked first in terms of the number of reported cases [12]. In addition, in 2018 two important outbreaks occurred in Italy that required increased environmental monitoring ([13]; data unpublished).

Most European countries have adopted a preventive approach, implementing actions for prevention and control of *Legionella* contamination. Monitoring *Legionella* contamination of potable water systems is of paramount importance for risk assessment. To this end, the plate culture method, performed using specific media (buffered charcoal yeast extract, BCYE), usually supplemented with different combinations of antimicrobial selective substances, is considered the gold standard for detection and enumeration of *Legionella* in water samples [14]. Culture can also be performed in accordance with ISO 11731:2017, an updated norm which replaced both ISO 11731:1998 (used in this study) and ISO 11731-2:2004. [15,16,17]. Although plate culture methods are specific for *Legionella*, they have high variability in enumeration, are time-consuming and require significant experience in recognizing *Legionella* colonies [18]. In addition, the enumeration of a *Legionella* concentration may be under-estimated due to the inability to detect viable but not-culturable bacteria or *Legionella* within amoebae [1]. Molecular methods based on the detection of *Legionella* DNA have been demonstrated to be highly specific and sensitive, are able to discriminate between species and serogroup and can detect viable but nonculturable bacteria, but are not considered fully suitable to enumerate *Legionella* in water samples because they are unable to reliably discriminate whether DNA detected is from live or dead organisms [19,20]. A promising alternative method is the Legiolert test (IDEXX Laboratories, Westbrook, ME, USA), a liquid culture method based on bacterial enzyme detection technology, which determines the most probable number (MPN) of exclusively *L. pneumophila* species present in water samples. The presence of *L. pneumophila* is visualized through the utilization of a substrate present in the Legiolert reagent. In published studies, Legiolert has shown equal performance to traditional plate culture methods, providing results in seven days with simplified sample preparation and analysis [21].

In this study, the detection and enumeration of *L. pneumophila* were determined in both 10 mL and 100 mL of potable water samples using the Legiolert method. Data obtained were compared with those obtained by the traditional plate culture method, performed according to the ISO 11731:1998 using 1 L of potable water, in order to evaluate the possibility of using the Legiolert method as a valid alternative to traditional plate culture. The results of this study are encouraging for the adoption of the Legiolert method for *L. pneumophila* enumeration in water samples.

## 2. Results

Overall, 123 potable water samples were analyzed. Fifty-one of them were positive by plate culture. Among these, 37 (30%) were *L. pneumophila*, typed as serogroups 1 or 2–14 positive, 14 (11.4%) were *Legionella* non-*pneumophila* and 58.6% were negative. Since the Legiolert test is designed for specificity for *L. pneumophila* only, the 14 samples that were positive with non-*pneumophila Legionella* species samples were excluded from further statistical comparisons with Legiolert results.

Legiolert test results derived from 10 mL water samples showed that 34/123 (27.6%) samples were positive for *L. pneumophila*, whereas results from 100 mL water samples detected 37/123 (30%) positive samples, consistent with plate culture results. Among the 37 plate culture positive samples, four showed very low *Legionella* concentrations (50, 150, 300 and 600 CFU/L) and these were found negative by the Legiolert test in both 10 and 100 mL water samples.

As per ISO 17994:2014 [22], eight samples that were positive with the Legiolert 100 mL test and exceeded the MPN count by Legiolert (too numerous to count, TNTC) were excluded from calculations. Thus, Legiolert data were analyzed for each of the 28 or 33 positive samples from the 100 mL and 10 mL tests, respectively, and were compared with plate culture data. Data analysis did not show significant differences in *Legionella pneumophila* detection between the two Legiolert protocols (*p* = 0.83), or between either Legiolert test (Legiolert 10 mL, *p* = 0.82; Legiolert 100 mL, *p* = 0.2) and the plate culture method performed according to ISO 11731:1998 (Table 1).

## 3. Discussion

Legiolert is characterized by very easy and rapid sample preparation, with the additional advantages of avoiding the need for large sampling volumes, membrane filtration, treatments, plating, colony isolation and additional confirmation or identification. Furthermore, the Legiolert test reduces the time required to obtain confirmed results (seven days, rather than 10 or more days required by the plate culture method). In this study, potable water samples were analyzed by both plate culture and Legiolert methods and no significant differences were found when comparing results. Furthermore, the results of the analyses carried out using only 10 mL of water samples showed that the Legiolert test was equally reliable using 10 mL of water as using 100 mL.

Although the plate culture method is the gold standard for the detection and enumeration of *Legionella* in water samples, different laboratories may choose to follow different procedures, depending on the expected *Legionella* concentration in the samples they process, or even for economic reasons, affecting the reliability of the data when the same sample is analyzed by different laboratories. The plate culture method should be performed by accredited laboratories, according to norms recognized by the country’s accreditation body. Among the methods, there are the ISO 11731:1998 method or the ISO 11731-2:2004 method (both of which have since been replaced by ISO 11731:2017); the 2007 American public health association (APHA) method; the Association Française de Normalisation (AFNOR) method NF T90-431:2018; or the U.S. Centers for Disease Control and Prevention (CDC) method [23,24,25]. For ISO 11731:2017 [15], depending on the matrix to be analyzed, the user may select from four methods, four treatments and four selective culture media, for a total of 14 possible procedural scenarios. Regardless of the method used, plate culture involves many steps and significant time requirements. *Legionella* monitoring, as part of the risk assessment analysis, concerns many different buildings such as hospitals, hotels, public offices and, in the near future, according to the revision of the European directive concerning potable water requirements, every potable water system. In this study, although we analyzed potable water samples according to ISO 11731:1998, the differences between this method and ISO 11731:2017 did not affect the recovery of *Legionella pneumophila*, since the plating of the samples on BCYE medium, as suggested by ISO 11731:2017, would only improve the recovery of *Legionella* non-*pneumophila* species, which were excluded from this investigation. The MWY medium, although not included in ISO 11731:1998, is suggested by ISO11731:2017, and was adopted for this study because it has been known for many years to be the best for the recovery of *Legionella* from drinking water samples [26].

The newly drafted European Drinking Water Directive seems to have taken into consideration the emergence of many of these newer methods in Annex III part A, leaving to the national bodies the opportunity to choose the methods they find most appropriate for the purposes they specify [27]. In addition, the Legiolert method has recently been NF (Norme Francaise)validated by AFNOR certification and also included in the UK’s Blue Book of validated test methods [28]. For many laboratories, the inclusion of testing in the Drinking Water Directive might lead to a large amount of work, time and financial expense. The Legiolert method may positively affect some of these difficulties, as well as those linked to the management of large volumes of water samples required for analysis by plate culture methods.

This study represents a confirmation of the reliability of the Legiolert method compared with the plate culture method, supporting conclusions from previous studies that documented the consistency of Legiolert for potable and non-potable water samples, analyzed according to ISO 11731:2004, which employs the filtration of 100 mL of water and the acid-treatment of filters which are directly placed on selective agar plates [16,29,30].

One limitation of the Legiolert method is that it is designed to detect only *Legionella pneumophila*, whereas other species remain undetectable. *Legionella pneumophila* is the most common species responsible for LD cases in Europe and, for this reason, in a few regions, such as France, Belgium, and the province of Quebec, Canada, it was decided to monitor only *Legionella pneumophila*, whereas in other countries, there is still a great debate on this matter.

Three fundamental factors can be identified in favor of monitoring exclusively for *Legionella pneumophila*. The first is risk—*L. pneumophila* is the species almost always cited in clinical cases and outbreaks and is the species most commonly found in the environment; the second is that laboratories may save time, human resources and money, and they can employ those saved resources to analyzing additional samples or locations, instead of identifying other *Legionella* species, which represent a much lower health risk; the third is that routinely monitoring only for the most pathogenic species of a bacteria is already an established practice. For example, *Pseudomonas aeruginosa* is routinely monitored, rather than all species of *Pseudomonas*.

At the same time, it is well known that other *Legionella* species are pathogenic to humans, although they represent a fraction of infections, with the exception of *Legionella longbeachae*, which is found in soil, rather than water, and is mostly detected in Australia and New Zealand. However *Legionella longbeachae* is beginning to be isolated also in EU/EEA, representing the 2.5% of isolated species in 2018 while other known and unknown species of *Legionella* were detected only in 3.3% of notified cases (European Legionnaires’ disease Surveillance Network annual meeting 2019, unpublished data). It should be noted that the identification of species other than *L. pneumophila* suffers from extensive use of the urinary antigen, which exclusively detects *Legionella pneumophila* serogroup 1, and from the medium used for the isolation of *Legionella*, which has historically been optimized for *Legionella pneumophila*. Therefore, many cases caused by other species might not be detected even by culture for this reason. Until a suitable medium for growing other *Legionella* species is developed, a routine PCR test in diagnosing human specimens, capable of distinguishing between *Legionella pneumophila* and other species, should be adopted in order to identify the real burden of Legionnaires’ disease, as already demonstrated in a few countries [31,32,33,34,35,36,37]. The results of these studies will be able to confirm the real incidence of infections caused by other *Legionella* species and consequently to address the choices on what should be the focus of monitoring in the environment.

The imminent introduction of the new drinking water legislation concerning the monitoring of an increasing number of water systems, however, will probably lead to streamlined choices aimed at reducing health risk by researching the most pathogenic and prevalent species present in the environment. Despite this, it must still be considered that for specific countries where other species of *Legionella*, such as *Legionella longbeachae*, are the prevalent in specific non-water matrices such as compost, and are an increasing cause of LD cases, Legiolert should not be utilized [1,31,35,37].

Concerning the enumeration of *Legionella pneumophila*, in this study, the most probable number did not provide any count in four of the 123 water samples, which instead tested positive by plate culture, though they were at lower concentrations of 50, 150, 300 and 650 CFU/L, respectively. For any Legiolert test, the limit of detection is 1 MPN, independent of the analyzed volume—a limit low enough to theoretically match that of plate culture. During this study, we used 100 CFU/L as the limit of detection for plate culture. We therefore suppose that the four samples which were negative in Legiolert but positive in plate culture could potentially be due to experimental errors. They could be, for example, faint colors of the wells not recognized as positive by the users. Unfortunately, these samples or isolates could not be tested again, as *Legionella* colonies were not kept for further investigations for detectability using the Legiolert test. The same bias can be considered for the eight TNTC samples tested by the 100 mL Legiolert protocol, which could have been included in the analysis if the original sample had been diluted or run with the 10 mL Legiolert protocol. No samples were found positive by Legiolert and negative by plate culture.

Although the data obtained showed that the two methods were comparable, a higher number of water samples at low *Legionella pneumophila* concentrations should perhaps be analyzed in order to assess any possible limitations with the Legiolert test.

In conclusion, Legiolert may be considered a valuable test for the detection and enumeration of *Legionella pneumophila* in potable water samples, and it can be used as a valid alternative to the traditional plate culture methods, especially considering the simplified protocol and the ability to employ smaller sample volumes to obtain the same quantification. Finally, it can be extremely useful when it is known that there is a prevalence of *Legionella pneumophila* in the water system under investigation.

## 4. Material and Methods

Over a 4-month period, 123 potable water samples were collected from hospitals, health care facilities for elderly people and industries located in 8 cities of the northern and central regions of Italy.

### 4.1. Enumeration of Legionella pneumophila and Legionella Spp. by ISO 11731:1998

Water samples were collected in 2-L bottles (1.5 L collected) and, after proper mixing, 1L of each sample was analyzed by the culture method according to ISO 11731:1998. Water samples were collected according to the protocol contained in the Italian guidelines for *Legionella* [38] and were stored at 5 °C ± 3 °C until they were delivered (within 24 h) to the Italian reference laboratory for *Legionella,* where all samples were analyzed.

The sample was filtered through 0.2-μm polycarbonate membranes and the membranes were transferred to 10 mL of the same sampled water and were solubilized by vortexing. From the concentrate, three MWY (Modified Wadowsky-Yee, Oxoid, Thermo Fisher Diagnostics Limited, Cheshire, UK) agar plates were inoculated by spread plating: one plate with 0.2 mL of the concentrated sample, one with 0.2 mL of the concentrated sample pre-treated with acid and one with 0.2 mL of the concentrated sample pre-treated by heating to 50 °C ± 1 °C for X 30 ± 2 min in a water bath. All the plates were then incubated at 36 °C ± 1 °C, with 2.5% CO_2_ for ten days. Presumptive *Legionella* colonies were confirmed by sub-culturing at least five colonies on BCYE agar plates with and without L-cysteine. The latex agglutination test (DR0800, Oxoid, Thermo Fisher Diagnostics Limited, Cheshire, UK) was utilized to obtain species information.

This study was carried out prior to the publication of the revision of the ISO 11731:2017 norm and the ISO 11731:1998 procedure was therefore applied. Regardless, the ISO 11731:1998 procedure adopted is still included in the new ISO 11731:2017 and is applicable for use with potable water samples, particularly when no information about the range of the *Legionella* concentration is known.

### 4.2. Enumeration of L. pneumophila by Legiolert/Quanti-Tray/Legiolert

The Legiolert test detects *Legionella pneumophila* through bacterial enzyme detection technology, which utilizes a substrate present in the Legiolert reagent in a liquid culture to reveal the presence of *L. pneumophila*. Generally, 100 mL of the culture is analyzed and results are received in 7 days. Any turbidity and/or brown color greater than the negative control indicates positivity. Enumeration is based on MPN. In this study Legiolert was performed using both 100 mL and 10 mL of the original 1.5 L water sample collected. Each aliquot was processed and analyzed following the procedure outlined in the Legiolert instructions, using the Quanti-tray/Legiolert device. Quanti-trays were incubated for 7 days at 39 °C +/−0.5 °C in a humidified environment.

### 4.3. Statistical Analyses

Data from plate culture and Legiolert testing were statistically analyzed by using Student’s *t*-test and with relative difference according to ISO 17994:2014 [22].

## Figures and Tables

**Table 1:** Comparison of Legiolert 10 mL and Legiolert 100 mL tests and plate culture according to ISO 17994:2014.

Comparison	Mean Relative Difference	95% CI		N.	Mean ± SD	*p*-Value
Legiolert 10 mL	0.13	−0.59	0.33	22	6.97 ± 2.10	0.8322
vs.						
Legiolert 100 mL					7.10 ± 1.79	
Legiolert 10 mL	−0.11	−1.58	1.35	33	6.94 ± 3.57	0.9042
vs.						
Plate Culture					7.05 ± 3.94	
Legiolert 100 mL	−0.73	−1.86	0.39	28	5.86 ± 3.02	0.3528
vs.						
Plate Culture					6.59 ± 2.83	
